# Development and content validity of the Person Experiences Interview Survey (PEIS): a measure of the mental health services experiences of people with developmental disabilities

**DOI:** 10.3389/fpsyt.2023.1271210

**Published:** 2023-11-30

**Authors:** Jessica M. Kramer, Joan B. Beasley, Andrea Caoili, Luke Kalb, Micah Peace Urquilla, Ann E. Klein, Janie Poncelet, Sandra Black, Richard C. Tessler

**Affiliations:** ^1^Department of Occupational Therapy, University of Florida, Gainesville, FL, United States; ^2^Institute on Disability, University of New Hampshire, Durham, NH, United States; ^3^National Center for START Services, Institute on Disability, University of New Hampshire, Durham, NH, United States; ^4^Kennedy Krieger Institute, Baltimore, MD, United States; ^5^University of Massachusetts Amherst, Amherst, MA, United States

**Keywords:** healthcare surveys (MESH term), developmental disabilities (MESH term), mental health services (MESH term), self-report, patient-reported experience measure

## Abstract

**Purpose:**

People with developmental disabilities and mental health service experiences have a right to be included in healthcare decisions, including the evaluation of their mental health services and providers. However, few self-report measures address this need. This study aimed to fill this gap by developing and evaluating the content validity, including comprehension, relevance, and comprehensiveness, of the Person Experiences Interview Survey (PEIS) with people with developmental disabilities and mental health experiences.

**Methods:**

The research team established a measurement framework based on the Family Experiences Interview Survey (FEIS), resulting in 21 PEIS items that were written in collaboration with young adults with developmental disabilities and mental health service experiences. Comprehension, relevance, and comprehensiveness were evaluated through cognitive interviews with people with developmental disabilities and mental health service experiences (respondents; *n* = 9) ages 23–49 years. Comprehensiveness and relevance were also evaluated in focus groups with family caregivers (*n* = 9) and mental health providers (*n* = 10) who serve this population. Two researchers independently coded open-ended responses to the PEIS for comprehension. A content validity index (CVI), indicating relevance, was calculated for each participant group for each item, and comprehensiveness was rated for item sets.

**Results:**

Fifteen of the 21 items met the criteria of ≥80% comprehension, with 89–100% of responses containing all or some intended information. All items met the CVI ≥80% criterion in at least two of the three groups. In all item sets, between 1 and 4 family members or providers felt one question was missing. Respondents used the response scale in a manner that corresponded with their open-ended descriptions, and family caregivers and providers had positive feedback about the response scale’s visual cues and number of choices. Using these findings, four items were removed and six items were revised, resulting in a 17-item measure.

**Conclusion:**

This study presents a novel and promising measure, the Person Experiences Interview Survey (PEIS). It also demonstrates that the employment of accessible methods allows people with developmental disabilities to meaningfully evaluate mental health services and providers. The PEIS shows great promise for application in the field by engaging those directly involved in the evaluation of mental health services and providers.

## Introduction

The evaluation of healthcare is predicated on the perspectives of people receiving those services. Person-reported measures are deployed to enhance the quality of care, inform both policy and practice, and improve clinical outcomes (1, 2). This is true for all people receiving care, including people with developmental disabilities, which include people with disabilities such as autism, cerebral palsy, and intellectual disability.^1^ However, there have been few, if any, measures of mental health service experiences for people with developmental disabilities. The reliance on proxy respondents is problematic in so far as it is grounded in stigma regarding both disabilities and mental health conditions. Assumptions that people with mental health conditions and developmental disabilities cannot serve as primary respondents because they are unstable, lack insight, and are easily influenced by others have led to discrimination in healthcare settings, particularly regarding a person’s preferences, needs, and goals (5, 6).

To overcome this narrative, disability advocates have called for ‘nothing about us without us’ in healthcare, and professionals have a responsibility to respect this call to action that underscores the autonomy of people with developmental disabilities (7, 8). One approach to operationalizing autonomy is to systematically provide opportunities for people with developmental disabilities to give input about their experiences with mental health providers and services. Such measures must be accessible and relevant to the needs and views of the informant, such as people with developmental disabilities (9, 10). A growing body of evidence demonstrates that when measures are designed to be cognitively accessible, people with developmental disabilities can provide quality responses and have the opportunity to share their perspectives about their preferences, needs, and goals (9, 11–13). Eliciting the direct perspectives of people with developmental disabilities is crucial, as research also demonstrates that proxy respondents such as family members may have perceptions and needs that are different from the person with the disability (14, 15).

Patient or person experience is one component of healthcare evaluation. Measures of experience, called patient-reported experience measures (PREMs), typically include communication, involvement in decision-making, information sharing, safety, comfort, efficiency, and respect (2, 16, 17). PREMs capture the extent to which these components of the healthcare experience occurred (16). PREMs are under the umbrella of measurement-based care, which requires the systematic administration of assessments to understand outcomes associated with clinical care (18). Those measures are then applied in a learning healthcare system, which is a healthcare organization that systematically integrates and evaluates its safety, quality, efficiency, and effectiveness based, in part, on patient-reported outcomes (19).

Little is known about the perspectives of people with developmental disabilities regarding their experiences with mental health providers and services. Ideally, a PREM for people with developmental disabilities and mental health service experiences would address three domains of service effectiveness, summarized as “the 3 A’s” (20). This includes access (services are timely and responsive), appropriateness (services match needs and wishes), and accountability (services have desired outcomes) (20).

Content validity is the most important property of self-reported health measures and the first step in measurement development (21, 22). Content validity includes item comprehension (understanding and interpretation), item relevance (meaningfulness), and the comprehensiveness (inclusiveness) of the item set. Therefore, content validity ensures that the measure captures what it is intended to measure. Content validity often requires the need for population-specific methods and measures to target specific components of service delivery (23). Importantly, content validity must be evaluated from the perspective of the intended reporters and users of the information. For self-reported measures about mental health services, this includes people with developmental disabilities, their family members, mental health providers, and administrators. For example, people with developmental disabilities may benefit from measures that use familiar language or that allow accommodations in the administration process to support cognitive processing (9, 10).

This study reports the initial development and evaluation of a new approach to measuring people with developmental disabilities’ experiences with mental health services and providers. The aim of this research study was to establish a valid and accessible measure for people with developmental disabilities to report their experiences with mental health services and providers. Beyond describing the development process, the research question was: What is the content validity of the Person Experiences Interview Survey (PEIS)? Specifically, are the items understood and answered in the intended manner (comprehension), and are they relevant and comprehensive for people with developmental disabilities who receive mental health services?

## Development of the Person Experiences Interview Survey

The PEIS was developed as a companion for the Family Experiences Interview Survey (FEIS) (24). The FEIS is applicable to any family caregiver who supports a loved one who has ongoing mental health service experiences and measures their experiences with their family members’ mental health services and providers from *their* perspective. Given that the FEIS has proven useful for clinical and scientific endeavors related to persons with developmental disabilities (25, 26), particularly the three sections (professional involvement, evaluation of client services, and quality of care), the research team aimed to develop a parallel self-report version for people with developmental disabilities. Similar to the FEIS, the PEIS was designed to be used by a broad group of people with developmental disabilities across adulthood with a variety of mental health service needs.

The development of the PEIS was an iterative process and followed three steps conducted in collaboration with people with developmental disabilities and mental health experiences: (1) measure conceptualization, (2) draft item development, and (3) item refinement. The leadership team consisted of experts in crisis prevention and intervention, outpatient mental health treatment, cognitively accessible patient-reported outcomes, measurement, survey development, and the lived experience of developmental disabilities and mental health experiences. In addition, the leadership team is the principal author of the FEIS.

### Measure conceptualization

The leadership team reviewed concepts from quality and mental health service evaluation frameworks (27–29). We aligned the PEIS questions with the three A’s framework (20). The first, access, is the ability to use inclusive mental health services in a timely fashion. The ability to receive services where and when they are needed is essential for effective service delivery. The second, appropriateness, is the ability of services and providers to address people’s specific needs. Mental health service providers must therefore have the capacity to diagnose and treat individuals with developmental disabilities using the optimal instruments, strategies, and approaches. Access alone does not benefit people with developmental disabilities if services and providers are not appropriate. The third effectiveness indicator, accountability, occurs when service providers evaluate outcomes, solicit input, and adapt in response. All three indicators require the engagement of people with developmental disabilities and their families to determine if the services are effective. While the PEIS items are not structured into subscales representing these indicators, they are grounded in this framework.

### Draft item development

The leadership team analyzed FEIS data from three sections (professional involvement, evaluation of mental health services, and quality of care) historically used by the START (Systemic, Therapeutic, Assessment, Resources, and Treatment) network (25, 26). START is a mental health crisis prevention and intervention service for people with developmental disabilities across the lifespan. Exploratory and confirmatory factor analyses (*n* = 1940) were employed to understand if the original factor structure was replicated when modified for family caregivers of people with developmental disabilities who had mental health needs. This analysis did not provide evidence for three separate subscales, nor did it suggest the removal of particular items to accommodate this model (unpublished data). As such, the leadership team developed the PEIS as an index where items are best evaluated at the individual level.

**Figure 1:**
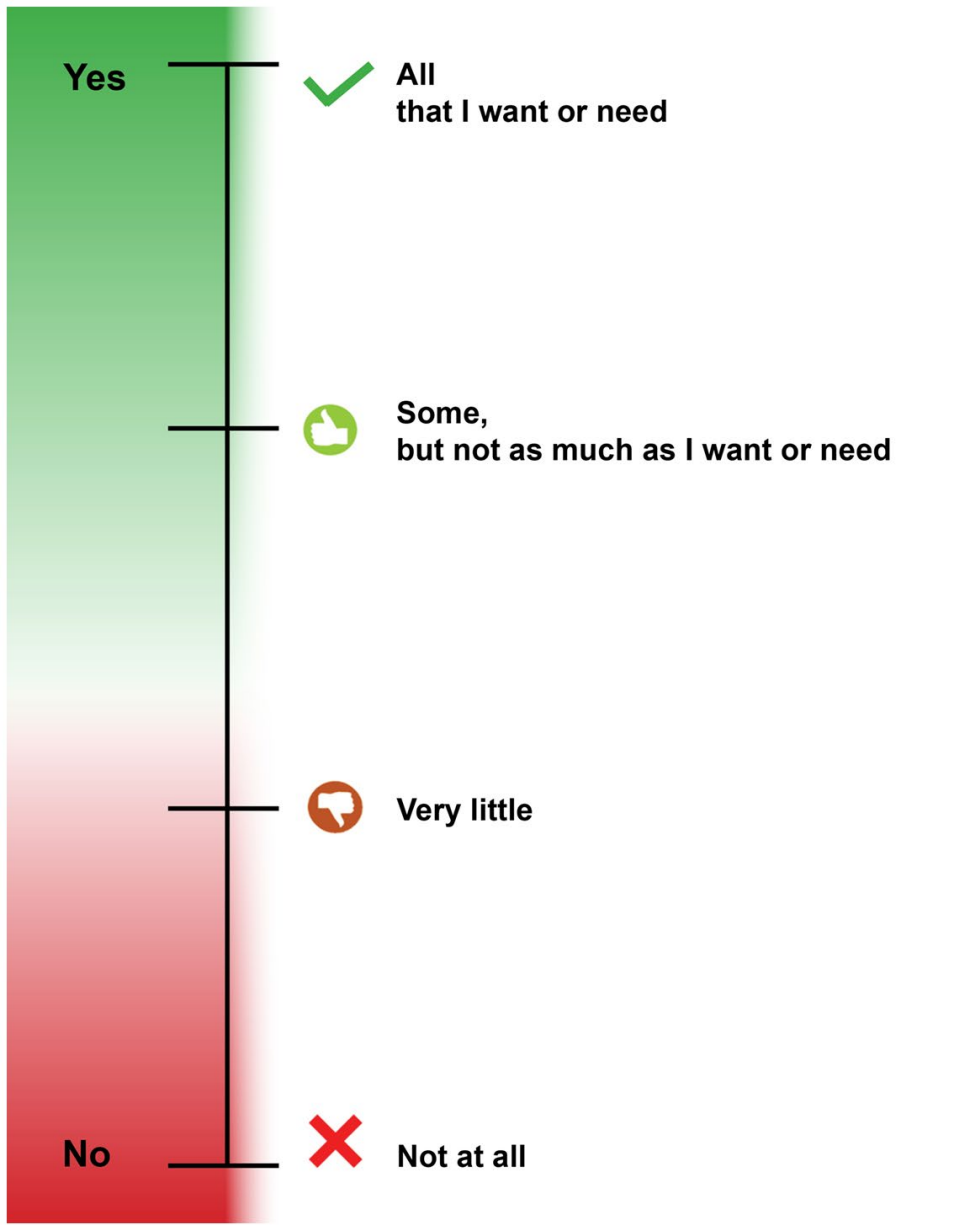
PEIS response scale with visual supports.

The leadership team drafted PEIS items from a one-to-one adaptation of each FEIS item, resulting in 22 draft items. The primary concept evaluated in each FEIS item was identified (e.g., treatment choices, who to contact when help is needed) and then translated into a cognitively accessible statement. Less-known words and phrases were replaced with those that are familiar to more people, and the total number of words per statement was also reduced, drawing upon our team’s previous experience designing cognitively accessible instruments (10, 12). The FEIS response scale words remained the same but were adapted to incorporate visual cues to facilitate the understanding of an ordered Likert scale (Figure 1). Response categories were organized vertically, with responses representing more perceived effectiveness at the top. The vertical continuum used progressive color shading to differentiate between Likert response categories, with green representing more perceived access, appropriateness, and accountability and red representing less to none. Each response was associated with a unique icon in a corresponding color, commonly associated with the concepts of endorsement or non-endorsement.

### Item refinement in partnership with people with lived experience of developmental disabilities and mental health service experiences

A PEIS workgroup was convened to review all draft items. The workgroup, co-facilitated by a researcher (first author) and an individual with lived experience (fifth author), was made up of three additional people who identified as having developmental disabilities and mental health service experiences. As reported elsewhere (masked for peer review), the workgroup reviewed each PEIS item for clarity and accessible language (e.g., avoiding long sentences and complex words) and provided feedback on the relevance of questions for people with disabilities. Items were revised and re-reviewed by the workgroup until no additional concerns were identified. One item was cut due to a lack of relevance. The workgroup also applied the response scale to each item to ensure the four categories and corresponding visual cues maintained a consistent meaning across items. Throughout this process, the workgroup identified three priorities when evaluating mental health services and providers: (1) clearly define mental health providers and services to be assessed, (2) ensure the respondent understands how their responses will be used and demonstrate that their perspective matters, and (3) provide accommodations to allow all people to provide their perspectives using the PEIS. These priorities were incorporated into the PEIS administration protocols. Following item refinement, qualitative methods were used to evaluate the content validity of the PEIS.

### Description of the PEIS

The PEIS allows people with developmental disabilities to evaluate their experiences with mental health services and providers and the extent to which they are easy to access, appropriate, and accountable. The version of the PEIS evaluated in cognitive interviews and focus groups, as described below, included 21 items; 19 items are answered using a 4-point response scale with visual supports: “All that I want or need”; “Some, but not as much as I want or need”; “Very little”; and “Not at all” (Figure 1). Two items are open-ended questions about mental health providers and services. Mental health services and providers evaluated in the PEIS include prescribers (including doctors, psychiatrists, and nurses), counselors and therapists, and crisis response services (including crisis response teams and crisis support provided by prescribers or therapists). Aligned with the FEIS, the PEIS includes a recall period of 1 year.

The PEIS was designed to be administered as a supported interview about mental health service experiences. Other studies with a broad patient population report that independently completed surveys produce generic and positive information, with interviews being more likely to elicit negative information (16). Interviews are more likely to elicit complete information from a wider range of those receiving services, as interviews reduce response bias. In addition, for people with developmental disabilities, interviews provide an opportunity to make accommodations and clarify the intended item’s meaning. For example, a list of standardized examples was created for each item in the instance that a question was difficult to understand; the examples were generated by the leadership team and the PEIS workgroup.

## Methods

Content validity (comprehensibility, relevance, and comprehensiveness) was evaluated with standardized procedures for self-reported health measures outlined by COSMIN (COnsensus-based Standards for the selection of health Measurement INstruments), an initiative of international multidisciplinary researchers with expertise in the development and evaluation of health outcome instruments (22). The process included cognitive interviews and focus groups with people with developmental disabilities, the primary respondent and user of the PEIS, and secondary users of the PEIS including family caregivers and mental health providers. All study procedures were reviewed and approved by the governing Institutional Review Board. All participants completed informed consent prior to participation; for respondents with developmental disabilities who had a legal guardian, both the legal guardian and the respondent demonstrated an understanding of study procedures, risks, and benefits, and the respondent had the final choice to participate in the study. All participants received compensation for their participation.

### Participants

Convenience sampling was used to recruit people with developmental disabilities, family caregivers, and mental health providers into professional mental health and advocacy networks in the United States. Purposeful recruitment was used to identify participants with diverse characteristics, including race, ethnicity, age, and disability. Across groups, participants were from the South/Southeast (46.4%), Northeast (17.9%), West (17.9%), and Southwest (7.1%) regions of the United States.

#### Respondents with developmental disabilities

Inclusion criteria were: (1) ages 14–54 years; (2) have a developmental disability, including autism, cerebral palsy, or an intellectual disability; (3) have the ability to engage in a conversation verbally, using American Sign Language, or using an Augmentative and Alternative Communication (AAC) device; and (4) actively receiving mental health services. The exclusion criteria included those who did not speak English. Nine respondents completed cognitive interviews, exceeding the COSMIN criteria of 7 or more respondents per item (21, 22). Respondents were 23–49 years old (M = 36.44 years, SD = 8.08), mostly lived in a residential community setting (55.6%), and reported receiving mental health services for 5 or more years (77.7%). All respondents communicated verbally. For more information, see Table 1.

**Table 1:** Demographics of research participants.

	Individuals with IDD-MH % (*n* = 9)	Family members % (*n* = 9)	Mental health professionals % (*n* = 10)
*Gender identity*			
Female	66.7% (6)	77.8% (7)	90.0% (9)
Male	33.3% (3)	22.2% (2)	10.0% (1)
Non-Binary	0% (0)	0% (0)	10.0% (1)
*Race*			
Asian	0% (0)	11.1% (1)	0% (0)
American Indian/ Alaskan Native	11.1% (1)	0% (0)	0% (0)
Black/African-American	22.2% (2)	11.1% (1)	40.0% (4)
White	55.6% (5)	66.7% (6)	50.0% (5)
Two or more races, or race not listed	11.1% (1)	11.1% (1)	10.0% (1)
*Ethnicity*			
Hispanic/Latinx/Spanish Origin	0% (0)	22.2% (2)	20.0% (2)
Non-Hispanic	100% (9)	77.8% (7)	80.0% (8)
*Educational Level*			
High school or less	11.1% (1)	0% (0)	0% (0)
High School Diploma	66.7% (6)	22.2% (2)	0% (0)
Associate Degree/Trade Certification	22.2% (2)	0% (0)	0% (0)
Bachelor’s Degree	0% (0)	44.4% (4)	10.0% (1)
Graduate Degree	0% (0)	22.2% (2)	90.0% (9)
*Years of experience with mental healthcare*			
1–4 years	22.2% (2)	11.1% (1)	30.0% (3)
5–10 years	11.1% (1)	11.1% (1)	40.0% (4)
> 10 years	66.7% (6)	77.8% (7)	30.0% (3)
Mental health condition (individual with IDD-MH)*			
Anxiety	77.8% (7)	88.9% (8)	–
Bipolar Disorder	11.1% (1)	11.1% (1)	–
Depression or other mood disorder	66.7% (6)	33.3% (3)	–
Obsessive Compulsive Disorder	11.1% (1)	55.6% (5)	–
MH condition not listed	0% (0)	22.2% (2)	–
Developmental disability (individual with IDD-MH)*			
ADHD	44.4% (4)	11.1% (1)	–
Autism	22.2% (2)	88.9% (8)	–
Cerebral Palsy	11.1% (1)	11.1% (1)	–
Intellectual Disability	66.7% (6)	66.7% (6)	–
Other	22.2% (2)	22.2% (2)	–

^*^IDD-MH: Intellectual and developmental disabilities and mental health service experiences.

#### Family caregivers

The inclusion criteria were solely being the family caregiver of a person with a developmental disability, ages 14–54, who received mental health services. The exclusion criteria included those who did not speak English. Nine family caregivers participated, who had family members with developmental disabilities ages 16–40 (*M =* 25.11 years, SD = 9.58), and who lived at home (66.7%) or in a community residential setting (33.3%). Almost all family members (88.9%) had 5 or more years of experience navigating mental health services with their family members. Two family caregivers were married to each other; they participated and rated PEIS items independently. One family caregiver was the guardian of a respondent who also participated in cognitive interviews. For more information, see Table 1.

*Mental health providers:* Inclusion criteria were at least 1 year of experience delivering mental health services to people with developmental disabilities. Ten mental health providers participated in the focus groups. Most providers (70%) had 5 or more years of experience working with people with developmental disabilities and were trained in the disciplines of social work (60.0%), education (10.0%), mental health counseling/family therapy (20.0%), and applied behavior analysis (10.0%). For more information, see Table 1.

### Procedures

#### Cognitive interviews

Cognitive interviews were used to evaluate the ease of understanding (comprehension), importance (relevance), and comprehensiveness. Cognitive interviewing is a gold standard methodology for instrument development (11, 22, 30), yet it may be inaccessible to individuals with developmental disabilities due to cognitive and communication demands (12). This study employed a modified cognitive interview process conducted over Zoom™ to enhance accessibility, including: concurrent thinking aloud (described below); using the screen share function to display one item at a time; displaying the response scale with visual cues next to each question; taking breaks when needed; and allowing the individual to bring a support person of their choice (a professional or family member) (11, 12).

Prior to the administration of the PEIS, each respondent was introduced to the purpose of the PEIS and reviewed plain language definitions of prescribers, therapists/counselors, and crisis response providers. The recall period of 1 year was established using the calendar method (e.g., “think back to the date 1 year ago, what happened around that time?”), and the interviewer, respondent, and supporter (if present) generated a list of all mental health providers and services received in the last year. This list was used during the administration. Respondents had an opportunity to practice using the PEIS response categories before answering the PEIS questions. They answered the low-risk question, “How often do you get to do your favorite activity?” and received support to use the response scale as needed.

The interviewer (first author) read each PEIS item out loud while showing the item and response scale and asked prompts to elicit more details about how and why the respondent selected their response (for example, “tell me more about when your providers did/not not…”) (11, 12). If the respondent indicated they did not understand the question or provided a response that was off-topic, the interviewer read the standardized example for each question. The use of standardized examples ensures consistency across respondents, which is necessary for accurate assessment, and reduces the risk that the administrator will bias or influence the respondents’ responses (10). As needed, the interviewer also provided 1-year recall prompts and reminders of the names of the services and providers from the past year. For respondents who completed the interview with a supporter, the supporter was restricted to providing examples of specific interactions with mental health providers and services and was instructed not to provide their perception of the experience. Immediately following the think-aloud discussion of each item, the respondent rated the importance of each item (not important, important, and very important); the importance response scale included stars (zero to two stars for each response) to enhance accessibility.

At the completion of the PEIS interview, respondents were asked if any questions about mental health services and providers were missing. Each PEIS rating, importance rating, and type of prompt given were recorded for each item on a data collection sheet. All interviews were recorded and transcribed verbatim.

#### Focus groups

Family caregivers and mental health providers participated in separate focus groups over Zoom™ to provide feedback about the importance (relevance) and comprehensiveness of the PEIS from their perspective of supporting people with developmental disabilities (21, 22). It is not appropriate to ask family members and providers to evaluate ease of understanding (comprehensibility), as they are not the intended respondent (21). At the beginning of the focus groups, facilitators reminded participants that they wanted to hear a range of perspectives and that it was not necessary to achieve group consensus. After learning about the purpose and organization of the PEIS, participants were presented with sets of PEIS items organized by general content. Participants used the Zoom poll feature to evaluate if each question asked about something important for them to know as a provider or family member using a 4-point response scale: not at all important, a little important, important, and very important. As a group, participants discussed their perspectives on the importance of the items in each set and provided examples from their experience. Participants were also asked if any questions were missing within each item set. After all item sets were reviewed, participants used Zoom polls to evaluate the response scale and the recall period. All focus groups, including polls, were recorded and transcribed verbatim.

### Analysis

#### Comprehension as evaluated by respondents with developmental disabilities

This study operationalized the comprehension of the items and rating scale obtained from the cognitive interview with respondents with developmental disabilities following previously analytical procedures (12). Two researchers independently coded open-ended responses to each item for comprehension using one of the three codes: the response provided information aligned with the item meaning as given in the standardized item examples (intended), the response provided information not aligned with item meaning (unintended), or the response included a mix of intended and unintended information. This coding approach acknowledges that people can share intended information about their mental health services with accommodation and practice. In addition, single-word responses (e.g., yes, no, and maybe) or responses that only repeated the response categories, even after prompting, were coded as limited information.

Using only the open-ended descriptions provided after prompting, researchers also independently coded the perceived experiences. Perceived experience is defined as the quality of or satisfaction with the situation or context described in each item. Three codes were applied: positive experiences (e.g., “I liked that” and “They really listened to me”), negative experiences (e.g., “I felt angry when they treated me like that”), or mixed (“My med nurse is so nice, but I do not like the psychiatrist”). Researchers independently coded 2–3 cognitive interviews at a time, with agreement reached for 75.5, 86.0, 88.4, and 88.9% of all coded data for each round of coding. After independent coding, researchers met to review discrepancies and reach consensus. PEIS response choices, importance ratings, and prompts used were entered for each response.

Descriptive statistics were calculated by item for comprehension; for each item, 80% of respondents should respond as intended for acceptable comprehension (12). To evaluate comprehension of the response scale, pivot tables were used to compare satisfaction codes and response scale choices. Appropriate response scale use is indicated by a pattern in which positive PEIS ratings were selected more often when open-ended descriptions of experiences were positive and in which negative ratings were selected more often when open-ended descriptions of experiences were negative.

Descriptive statistics were also calculated for the number of times assistance was provided during administration (e.g., the respondent asked for clarification and/or the administrator read the standardized example or provided a reminder about the 1-year recall period). All percentages were calculated based on the total number of coded responses. Overall, there were very few responses that were missing or unable to be coded (*n* = 1, 1, and 2 for PEIS rating scale response, importance rating, and comprehension code, respectively); perceived experience codes had the highest level of missingness, with 14.8% of responses (*n* = 28) that were unable to be coded.

#### Relevance

To examine relevance, a content validity index (CVI) represented the percentage of respondents, family caregivers, and providers rating each item as “Very important” or “Important.” CVI ≥ 80% is the gold standard for acceptable relevance (31).

#### Comprehensiveness

To evaluate comprehensiveness from the perspective of respondents with developmental disabilities, we reviewed open-ended feedback to identify any missing content or questions. To evaluate comprehensiveness from the perspective of family caregivers and providers, we calculated the percentage of participants who identified missing content from the four-item sets (no missing questions, one missing question, and a few missing questions).

### Other feedback

Descriptive statistics were calculated for feedback about the potential intrusiveness of PEIS questions, the PEIS recall period, and the response scale.

## Results

### Item and response scale comprehension: respondents with developmental disabilities

Fifteen of the 21 items met comprehension criteria, with 89–100% of responses containing all or some intended information (Table 2). No item had less than 75% of responses containing all or some intended information. Across all items, 56.15% of responses provided only the information intended by the item, with an additional 33.16% of responses providing both intended and unintended information (a total of 89.30% of responses containing intended information). Across all items, very few responses contained only unintended (3.21%) or limited (7.49%) information. Of the 87 times that responses included unintended content, 61 referenced situations not aligned with the specific question, 4 were recalling situations longer than 1 year ago, and 6 were thinking of providers and services not included in the PEIS. Six respondents provided all or some intended information for all of their responses, with the remaining three having 4.75–15% of their responses containing only unintended information. Of those three, one respondent provided limited information on 45% of the items.

**Table 2:** Item comprehension and supports provided for tested items.

Tested PEIS question	Coded responses (*n* = 9)				Sum of responses with intended information	% of responses with administrator support	Final decision
	Intended information only	Intended and unintended information	Unintended information only	Limited response			
How much information did you get from your mental health providers about your mental health needs?	33.33%	44.44%	11.11%	11.11%	77.78%	66.67%	Cut: Limited comprehension
How much information did you get from mental health providers about how to help yourself feel better if you have a mental health crisis?	44.44%	55.56%	0.00%	0.00%	100.00%	55.56%	Revised to improve comprehension
How much information did you get from mental health providers about who to call if you have a mental health crisis	66.67%	33.33%	0.00%	0.00%	100.00%	22.22%	Revised to improve comprehension
How often did mental health providers give you a chance to make decisions about your treatment?	66.67%	22.22%	0.00%	11.11%	88.89%	44.44%	No changes
How often did your mental health providers help you with something you were worried about?	77.78%	22.22%	0.00%	0.00%	100.00%	22.22%	No changes
How much did mental health providers listen to your ideas about your mental health treatment?	33.33%	66.67%	0.00%	0.00%	100.00%	88.89%	Cut: Limited comprehension, repetitive content
How much did mental health providers understand how hard it can be to get mental health services?	12.50%	62.50%	0.00%	25.00%	75.00%	44.44%	Cut: Limited comprehension
How much do you meet with, talk to, or message with your mental health provider?	55.56%	44.44%	0.00%	0.00%	100.00%	22.22%	No changes
How often did you get the mental health services you needed?	44.44%	44.44%	0.00%	11.11%	88.89%	22.22%	No changes
How often did mental health providers ask you how much you like your mental health services?	77.78%	11.11%	11.11%	0.00%	88.89%	33.33%	No changes
How often did you get to choose your mental health services?	33.33%	55.56%	11.11%	0.00%	88.89%	55.56%	Cut: Limited comprehension
How often did you get to choose the person who provides your mental health services?	66.67%	22.22%	0.00%	11.11%	88.89%	44.44%	No changes
How often were mental health services provided at a time and place that was easy to get to?	37.50%	62.50%	0.00%	0.00%	100.00%	66.67%	No changes
How often did mental health services change to meet your needs?	55.56%	22.22%	11.11%	11.11%	77.78%	55.56%	No changes
How often were you happy with your mental health services?	66.67%	11.11%	22.22%	0.00%	77.78%	22.22%	Revised to improve comprehension
How much do you feel that mental health providers pay attention to people with needs like yours?	33.33%	44.44%	0.00%	22.22%	77.78%	66.67%	Revised to an open-ended question
How often do you get to say what you want or need for your mental health services?	55.56%	33.33%	0.00%	11.11%	88.89%	66.67%	No changes
How often were you happy with your family members’ involvement in your treatment?	88.89%	0.00%	0.00%	11.11%	88.89%	55.56%	Revised to improve comprehension
If you have a mental health crisis, how much help can you get at night or on the weekend?	77.78%	11.11%	0.00%	11.11%	88.89%	55.56%	No changes
Was there any service that you needed that was not available? What?^¥^	77.78%	0.00%	0.00%	22.22%	77.78%	0.00%	Revised to improve comprehension
What advice would you give to mental health providers? ^¥^	66.67%	33.33%	0.00%	0.00%	100.00%	0.00%	No changes
Total % responses	56.15%	33.16%	3.21%	7.49%	89.30%	48.24%	–

^*^n = 8 response. All percentages are out of the total available data, missing data excluded. ^¥^Open-ended question; response scale not used.

Across all items, 48.24% of responses were provided with support from the administrator. Varying levels of support were needed by item; 22.2–88.9% of responses required reminders of the recall period, the types of mental health services and providers to assess, and help to read standardized item examples.

Respondents used the higher response choices most often. “All that I want or need” was used 66.4% of the time, and the lower two response choices were used 12.6% of the time. However, there was a logical, observed correspondence between open-ended descriptions of experiences and response scale choice (Table 3). When descriptions of mental health providers and services were negative, 100% of responses used the lower two response choices. When open-ended descriptions of mental health providers and services were positive, all but 1 response selected the top two response choices (98.9%).

**Table 3:** Correspondence between PEIS response choice and open-ended description of experiences with mental health providers and services.

Open-ended description of experiences % (*n*)	PEIS Response Choice			
	Not at all	Very little	Some, but not as much as i want or need	All that i want or need
Negative experiences	33.3% (4)	55.7% (8)	0% (0)	0% (0)
Mixed experiences	2.6% (1)	10.5% (4)	65.8% (25)	21.1% (8)
Positive experiences	1.1% (1)	0% (0)	5.4% (5)	93.5% (87)
Total responses for each rating scale category % (*n*)	4.2% (6)	8.4% (12)	21.0% (30)	66.4% (95)

All percentages are out of the total available data, missing data excluded.

### Item relevance

All respondents with developmental disabilities rated the PEIS items as “important” or “very important” (Table 4). Mental health provider content validity indices (CVIs) ranged from 50 to 100%, with an average of 88%. Family caregiver CVI ranged from 77.8 to 100%, with an average of 94.% All items met the CVI 80% criterion in at least two of the three groups.

**Table 4:** Item relevance: content validity index (CVI)* for tested items.

Tested PEIS question	Individuals with IDD-MH (%)	Family members (%)	Providers (%)
How much information did you get from your mental health providers about your mental health needs?	100	88.9	100
How much information did you get from mental health providers about how to help yourself feel better if you have a mental health crisis?	100	100.0	100
How much information did you get from mental health providers about who to call if you have a mental health crisis	100	88.9	100
How often did mental health providers give you a chance to make decisions about your treatment?	100	100.0	100
How often did your mental health providers help you with something you were worried about?	100	100.0	80
How much did mental health providers listen to your ideas about your mental health treatment?	100	88.9	100
How much did mental health providers understand how hard it can be to get mental health services?	100*	77.8	90
How much do you meet with, talk to, or message with your mental health provider?	100	77.8	80
How often did you get the mental health services you needed?	100	100.0	90
How often did mental health providers ask you how much you like your mental health services?	100	77.8	80
How often did you get to choose your mental health services?	100	88.9	80
How often did you get to choose the person who provides your mental health services?	100	88.9	50
How often were mental health services provided at a time and place that was easy to get to?	100	100.0	90
How often did mental health services change to meet your needs?	100	100.0	80
How often were you happy with your mental health services?	100	88.9	80
How much do you feel that mental health providers pay attention to people with needs like yours?	100	100.0	100
How often do you get to say what you want or need for your mental health services?	100	100.0	90
How often were you happy with your family members’ involvement in your treatment?	100	88.9	80
If you have a mental health crisis, how much help can you get at night or on the weekend?	100	100.0	100
Was there any service that you needed that was not available? What?^¥^	100	–	–
What advice would you give to mental health providers? ^¥^	100	–	–

CVI = Percentage of participants rating item as important and very important/total number of participant responses. *n = 8. All percentages are out of the total available data, missing data excluded. ^¥^Family members and providers did not rate the importance of the open-ended questions.

### PEIS comprehensiveness

Most respondents with developmental disabilities said no questions were missing (Table 5). Some suggested adding questions specific to peer support (*n* = 1); ableism/stigma (*n* = 2); accommodations (*n* = 1); and medications (*n* = 1). In all item sets, between 1 and 4 family caregivers (11.1–44.4%) felt 1 question was missing, and between 1 and 3 providers (10–30%) felt 1 question was missing. Families suggested adding questions about medication choices (*n* = 1), feeling comfortable with the provider (*n* = 1), the ability to stop or refuse treatment (*n* = 1), addressing crises in a timely fashion (*n* = 3), role of family involvement (*n* = 5), and locating services (*n* = 1). Providers suggested adding questions about accessible information (*n* = 2), overall service effectiveness (*n* = 1), evaluating previous experiences (not current experiences, *n* = 1), the role of family involvement (*n* = 3), insurance (*n* = 1), provider preferences (*n* = 2), and other types of therapeutic approaches (e.g., outdoor therapy, *n* = 1).

**Table 5:** PEIS comprehensiveness of tested item set.

PEIS tested item set	Providers* *n* = 10	Family members* *n* = 9
Information	80%	55.6%
Input and control	90%	88.9%
Family involvement	50%	55.6%
Evaluation of services and providers	70%	66.7%

*Percentage of participants who rated each item set as complete (no more important question missing). Respondents with developmental disabilities did not provide ratings for comprehensiveness.

### Other feedback about the PEIS

No respondents with developmental disabilities felt the questions on the PEIS were too intrusive or private, and all liked the visual tool for the response scale. Providers and family caregivers liked the colors and visual cues of the response scale, and 90.0% of providers and 88.9% of providers felt the number of choices was “just right.” Providers and family caregivers diverged in their opinions about the 1-year recall period; some reported it was “just right” (60.0% providers, 44.4% family caregivers), “too long” (40.0% providers, 22.2% family caregivers), or “too short” (33.3% family caregivers). Both groups acknowledged that people needed time to establish services and a relationship with providers prior to evaluating services and that evaluations and planning often occur annually.

### Final PEIS

As a result of the content validity evaluation, four items were cut due to poor comprehension, and six items were revised to enhance comprehension. The resulting 17-item PEIS (with three questions using an open-ended format) is shown in Table 6.

**Table 6:** PEIS items after content validity results.

PEIS question
1. How much do you meet with, talk to, or message with your mental health provider?
2. How often did your mental health providers help you with something you were worried about?
3. How often did you get to say what you want or need for your mental health services?
4. How often did mental health services change to meet your needs?
5. How often did mental health providers ask you how much you like your mental health services?
6. How often did you get the choose the person who provides your mental health services?
7. How often did mental health providers give you a chance to make decisions about your treatment?
8. How often were you satisfied with your family member’s involvement in your treatment? *If you do not have a family member involved, you can think about a friend or other support person who is involved in your treatment*.
9a. How often were mental health services provided at a time and a place that was easy to get to? 9b. What made it hard to get services: (a) services were too far away (b) hard time getting transportation to services (c) could not get an appointment at a time that worked with my schedule.
10. How often did you get all the mental health services you needed?
11. If you had a mental health crisis, how much information did providers give you about how to feel better?
12. If you had a mental health crisis, how much information did you get about who to call?
13. If you have a mental health crisis, how much help can you get on nights or weekends?
14. How often were you satisfied with your mental health services?
15. Do mental health providers and services pay attention to the needs of people with disabilities? Why do you say that?*
16. Did you want any service that you could not get? What was it?*
17. What advice would you give to mental health providers?*

*Open-ended question, response scale not used.

## Discussion

This study details the development and content validity of the PEIS, a self-report measure of mental health service experiences for people with developmental disabilities This study fills an important gap as few-to-no measures address this topic from the service users’ experience, despite estimates that 40% of people with developmental disabilities have a mental health condition (32). Content validity was assessed as item comprehension, item relevance, and the comprehensiveness of items (21, 22). This study demonstrates that, with tools to support the process, people with developmental disabilities can meaningfully evaluate the extent to which their mental health services and providers are easy to access, appropriate, and accountable to their needs. The availability of tools such as the PEIS is one way for the field to build capacity to respond to the previous systematic exclusion and stigma of people with developmental disabilities (6, 8, 9).

Item comprehension is a crucial component of content validity for two reasons. First, items would simply be invalid if items were not understood as designed. Second, consistent interpretation of item meaning makes it possible to compare participant responses across populations to evaluate mental health service experiences (18, 19). On average, respondents with developmental disabilities responded to PEIS items with all or some intended information 88.3% of the time. The PEIS is designed to be administered as an interview with support and is not an independent questionnaire. The PEIS administration protocol incorporates standardized examples, an accessible response scale, and tools to remind respondents of the one-year recall period and the types of services and providers assessed. The intent is to allow administrators to provide support for people with developmental disabilities so they may share meaningful and relevant information about their mental health providers and service experiences. Throughout the process, respondents stated that they were grateful for the opportunity to share their perspectives.

Respondents with developmental disabilities consistently selected response choices that were logically aligned with their perceptions, further supporting comprehension. This aligns with other research using self-reported health measures with people with disabilities, in which rating scales developed to be accessible can be used appropriately (12, 33, 34). Respondents expressed satisfaction with their mental health providers and services, with 66.4% of responses indicating that they received “all that I want or need” across items. Importantly, 33.6% of responses still indicated that the range of mental health services and providers they interfaced with met their needs only some of the time, very little, or not at all. During the interviews, respondents expressed frustration with the limited availability of qualified providers, services that were not accessible for people with disabilities, and providers who did not listen to their concerns and preferences. This is a common theme in the literature, with a recognized shortage of providers qualified to provide effective mental healthcare to people with developmental disabilities (35, 36).

The CVI ratings across all three groups demonstrated the relevance of the PEIS items. This allows clinicians to better respond to explicit issues faced by many receiving mental health services and provides the opportunity to improve the access to, appropriateness, and accountability of those services. It also gives researchers the opportunity to evaluate trends in mental health service experiences overall as reported by people with developmental disabilities.

The 21 items within the PEIS provide a comprehensive measure of mental health service experiences. While all three groups suggested content to add, most of the content was already explicitly or implicitly addressed in the existing items. For example, references to medication choice and control were included in the standardized examples for five items, and ableism/stigma were indirectly addressed in one question. Given the potential duplication of content, additional questions were not added to ensure the PEIS could be completed within a feasible length of time. Some content recommended by participants was not aligned with mental health services widely available to people with developmental disabilities or with content on the FEIS, for example, peer support. It is recommended that the FEIS continue to be used to evaluate family caregiver experiences, as they offer a unique and important perspective. A comparison of the two perspectives may be informative going forward.

Further evaluation of the PEIS is needed to understand its item and test characteristics. Descriptively, item distributions should be assessed to understand floor and ceiling effects. Items should also be assessed for sensitivity to change, perhaps trialed as an outcome measure for an intervention study. Fortunately, the authors of this study will be using the PEIS for this purpose. For validity, we recommend evaluating construct validity to understand if the PEIS is measuring a similar concept as similar measures (e.g., using established satisfaction scales). For reliability, we recommend test–retest and Cronbach’s alpha as a measure of temporal, construct, and internal consistency. The PEIS will not be fully ready for wider use in the field until such metrics are demonstrated.

This study has several limitations. Although the number of participants in each group exceeds the minimums recommended by COSMIN (21), the experiences of the participants in this study may not be shared by all people with developmental disabilities, their family caregivers, and their providers. For example, people who use AAC were not enrolled in this study. Additionally, while the targeted inclusion age for respondents with developmental disabilities and family caregivers was the same across groups, there were slight differences in the age range and average age across the two participant groups. Thus, the generalizability of this study is limited. In addition, diagnoses were not independently verified with medical records or formal screening tools, and we did not measure the functional or communication level of respondents with developmental disabilities. However, as the participants in this study had many years of experience with mental healthcare, it is likely that respondents with disabilities and their family caregivers had experience advocating about their disability and mental health and were likely trustworthy reporters. Finally, extensive psychometric testing could not be applied, as this study was designed to develop and assess the content validity of the PEIS.

## Conclusion

Providing access to the patient-reported evaluation of mental healthcare is crucial if the field is to live out the mantra ‘nothing about us, without us’ and reduce stigma in mental healthcare (6, 8). Developed in collaboration with people with lived experiences, the PEIS appears to be one content-valid approach for people with developmental disabilities to evaluate their experiences with mental health services and providers and the extent to which they are easy to access, appropriate, and accountable. Furthermore, the cognitive interviews and qualitative coding approach used in this study demonstrate that people with developmental disabilities can evaluate their mental health and services when using measures designed to be cognitively accessible. Future research is needed to understand the measures’ psychometric properties and application in clinical services.

## Data availability statement

The original contributions presented in the study are included in the article/supplementary material, further inquiries can be directed to the corresponding author.

## Ethics statement

The studies involving humans were approved by University of New Hampshire IRB. The studies were conducted in accordance with the local legislation and institutional requirements. Written informed consent for participation in this study was provided by the participants' legal guardians/next of kin.

## Author contributions

JK: Conceptualization, Formal analysis, Funding acquisition, Investigation, Methodology, Supervision, Writing – original draft. JB: Conceptualization, Funding acquisition, Methodology, Project administration, Supervision, Writing – original draft. AC: Conceptualization, Formal analysis, Methodology, Writing – original draft. LK: Conceptualization, Funding acquisition, Methodology, Supervision, Writing – original draft. MU: Conceptualization, Formal analysis, Writing – review & editing. AK: Conceptualization, Writing – review & editing. JP: Conceptualization, Formal analysis, Writing – review & editing. SB: Formal analysis, Writing – review & editing. RT: Conceptualization, Writing – review & editing.

## Footnotes

^1^The Developmental Disabilities Assistance and Bill of Rights Act of 2000 defines a developmental disability as a condition that is attributable to a mental and/or physical impairment, manifested before the age of 22, long-term, results in significant limitations in multiple areas of functioning, and requires specialized supports (3). Developmental disabilities reflect a broad range of conditions, such as autism spectrum disorder, intellectual disability, and cerebral palsy (4).
